# Supplementary material for: Socioeconomic determinants of virtual care use among people living with HIV in a clinical cohort in Ontario, Canada: A cross-sectional study
Source: PLoS One. 2026 Feb 19;21(2):e0326989. doi: 10.1371/journal.pone.0326989 (PMC12919790; doi:10.1371/journal.pone.0326989)
Supplement: S1 File — (DOCX) [file pone.0326989.s004.docx]

**STROBE Checklist for Cross-Sectional Studies**

Instructions: Please use this checklist to ensure all relevant items of the STROBE guidelines for cross-sectional studies are addressed in your manuscript. Tick the box once each item is completed.

| Section | Item No. | Recommendation | Tick |
| --- | --- | --- | --- |
| Title and Abstract | 1 | Indicate study design in the title or abstract. | [✓ ] |
|  | 2 | Provide an informative and balanced summary of what was done and found. | [✓] |
| Introduction | 3 | Explain the scientific background and rationale for the study. | [✓] |
|  | 4 | State specific objectives and hypotheses. | [✓] |
| Methods | 5 | Describe key elements of study design early in the paper. | [✓] |
|  | 6 | Describe the study setting, locations, and relevant dates. | [✓] |
|  | 7 | Give eligibility criteria, sources, and selection methods of participants. | [✓] |
|  | 8 | Clearly define outcomes, exposures, predictors, confounders, and effect modifiers. | [✓] |
|  | 9 | Describe sources of data and methods of assessment. | [✓] |
|  | 10 | Describe any efforts to address potential sources of bias. | [✓] |
|  | 11 | Explain how study size was determined. | [✓] |
|  | 12 | Explain how quantitative variables were handled in the analysis. | [✓] |
|  | 13 | Describe all statistical methods, including confounding control, subgroup analyses, and sensitivity analyses. | [✓] |
| Results | 14 | Report numbers at each stage; reasons for non-participation. | [✓] |
|  | 15 | Provide characteristics of participants; exposures and potential confounders. | [✓] |
|  | 16 | Report number of outcome events or summary measures. | [✓] |
|  | 17 | Give unadjusted and adjusted estimates with precision; clearly state which confounders were adjusted for. | [✓] |
|  | 18 | Report subgroup analyses, interactions, and sensitivity analyses. | [NA] |
| Discussion | 19 | Summarize key findings with reference to study objectives. | [✓] |
|  | 20 | Discuss limitations, potential bias, and their direction and magnitude. | [✓] |
|  | 21 | Provide a cautious overall interpretation of results considering objectives, limitations, multiplicity of analyses, and other evidence. | [✓] |
|  | 22 | Discuss external validity or applicability of the findings. | [✓] |
| Other Information | 23 | Describe sources of funding and role of funders. | [✓] |
|  | 24 | State ethics approval and consent procedures. | [✓] |
