# Supplementary material for: Socioeconomic determinants of virtual care use among people living with HIV in a clinical cohort in Ontario, Canada: A cross-sectional study
Source: PLoS One. 2026 Feb 19;21(2):e0326989. doi: 10.1371/journal.pone.0326989 (PMC12919790; doi:10.1371/journal.pone.0326989)
Supplement: Attachment — Submitted filename: Response to Reviewers.docx [file pone.0326989.s006.docx]

**Nadia Rehman,**

PhD candidate,

Department of Health Research Methods, Impact, Evidence, and Impact,

1280 Main Street West,

Hamilton, ON L8S 4K1

Tel: 289-439-7433

Email: [Nadia Rehman](mailto:Nadia%20Rehman)

November 15^th^, 2025

**Subject: Appeal and Revised Submission – Manuscript ID PONE-D-25-31000**

Dear PLOS ONE Editorial Team,

Thank you for the opportunity to appeal the rejection decision of our manuscript (PONE-D-25-31000), titled “Socioeconomic determinants of virtual care use among people living with HIV in a clinical cohort in Ontario, Canada: A cross-sectional study,” for reconsideration in PLOS ONE.

We sincerely appreciate PLOS ONE for giving us another opportunity to revise the manuscript, and we are grateful to the reviewers for their time and thoughtful feedback. We have carefully addressed each of their comments and incorporated the suggested changes to enhance the clarity, quality, and scientific rigor of our manuscript.

Below, we provide a detailed, point-by-point response to all comments. The revised manuscript and a marked-up version highlighting the changes are attached. We believe these revisions align with the journal’s standards and hope they meet the expectations of both the reviewers and the editorial team.

Sincerely,

Nadia Rehman

| **Reviewer’s Comments** | **Authors’ Response** | **Page No.** |
| --- | --- | --- |
| **Major response: Have the authors made all data underlying the findings in their manuscript fully available?** | | |
| 3. The PLOS Data policy requires authors to make all data underlying the findings described in their manuscript fully available without restriction, with rare exception (please refer to the Data Availability Statement in the manuscript PDF file). The data should be provided as part of the manuscript or its supporting information or deposited to a public repository. For example, in addition to summary statistics, the data points behind means, medians and variance measures should be available. **If there are restrictions on publicly sharing data—e.g. participant privacy** or use of data from a third party—those must be specified. | We thank the reviewers for this comment. We appreciate the importance of PLOS ONE’s data availability policy and have revised the manuscript accordingly.  However, the authors cannot share the dataset directly, as it was obtained under a data-use agreement with the Ontario HIV Treatment Network (OHTN) through the Ontario HIV Treatment Cohort Study (OCS). Access to the data required an extensive review process, including evaluation of the study protocol by eight reviewers, followed by final approval from the OCS Governance Committee. This approval was granted after the protocol was presented at a meeting and after agreement to adhere strictly to the study’s privacy, confidentiality, and dissemination policies. These agreements include strict restrictions that prevent public distribution of the data in any form. However, the data are available to qualified researchers upon request through the OHTN’s Research Application Process (RAP).  We have now included a detailed Data Availability Statement in the manuscript. We previously understood that this information had to be provided only in the application; however, to fully address the reviewer’s concern and ensure transparency, we have now added these details to the manuscript as well.  The following statement has been added to the manuscript:  Data Availability Statement:  “The data used in this study were obtained from the Ontario HIV Treatment Network Cohort Study (OCS). Due to privacy and confidentiality agreements, the authors cannot share the dataset or raw data publicly. However, data access may be requested directly from the OHTN by submitting a [Research Application Process (RAP) form.](https://ohtncohortstudy.ca/research/) Requests can be made by emailing <ocs@ohtn.on.ca>  .” | **Page 23, Lines 328-332** |
| **Response to Reviewer #1** | | |
| 1.This manuscript provides a detailed overview of Socioeconomic determinants of virtual care use among people living with HIV in a clinical cohort in Ontario, Canada : A cross-sectional study. However, it contains a few errors. The manuscript, in its current form, requires significant improvements. Here are some detailed comments: Is the choice of cross-sectional design appropriate for answering the research question of this study ? | We thank the reviewer for this comment. This study represents an initial evaluation of the associations between sociodemographic variables and choice of care modality among people living with HIV. Our cross-sectional analysis using multinomial logistic regression identifies statistically significant trends for factors such as gender, region, education and people with depression, indicating that these variables influence whether participants choose virtual or in-person care.  Beyond identifying associations, these findings have important public health and policy implications. Understanding why some individuals prefer one modality over another allows us to assess whether virtual care is equitably accessible to all populations. Equitable access is critical, as progress toward the UNAIDS 95-95-95 targets is hindered by sociodemographic and structural barriers. This work provides foundational insights into potential disparities in care utilization and informs policymakers in developing strategies to improve access and uptake of virtual care among diverse groups of people living with HIV.  While causal inference is not possible with a cross-sectional design, this approach is appropriate for identifying associations and generating hypotheses for future longitudinal, interventional, and qualitative studies, which can further explore how to maximize the benefits of virtual care and reduce barriers to HIV care. |  |
| 2. Do you consider the socio-economic level measure to be relevant? If so, please explain why. | We thank the reviewer for this comment. Engagement in the HIV care cascade remains below the UNAIDS 95-95-95 targets in many populations. Virtual care represents one potential strategy to reduce barriers to care, particularly for socioeconomically disadvantaged groups. However, without systematically evaluating its impact and effectiveness, virtual care interventions may inadvertently exacerbate disparities rather than provide benefits. Including socioeconomic measures allows us to assess whether virtual care is equitably accessible and identify populations at risk of being left behind, ensuring that interventions are both effective and inclusive. |  |
| 3. This study sought to understand the factors that influence the types of care modalities used, as this information can help remove barriers to care retention. At the end of this work, can we confirm that the objective was achieved? If so, add a paragraph on the contribution of this work to public health. | We thank the reviewer for this comment. Yes, the objective of our study—to understand the factors influencing the choice of care modality among people living with HIV—was achieved. Our analysis identified statistically significant associations between sociodemographic factors, including gender, region, education, and depression, and the use of virtual versus in-person care. These results highlight patterns in care preferences that can inform targeted interventions.  “Virtual care can advance the UNAIDS 95‑95‑95 targets by improving access, retention, and adherence to HIV care, particularly for populations facing geographic, socioeconomic, or mobility barriers [39]. Understanding patterns of virtual care use allows healthcare systems to identify underserved groups and implement equity-focused strategies [40]. By complementing in-person services, virtual care supports patient-centred, differentiated care, strengthening the public health response to HIV [38].” | **Page 21, Lines 285-289** |
| 4. The study has limitations that may affect the interpretation of the results. In light of this observation, what corrective measures do you envisage to mitigate this major limitation? | We thank the reviewer for this observation. We acknowledge that, as a cross-sectional study, causal inference is not possible and that unmeasured confounding or selection bias may influence the observed associations. Additionally, reliance on self-reported data for certain variables could introduce reporting bias.  To mitigate these limitations, we employed adjusted multinomial logistic regression to account for multiple relevant sociodemographic and clinical covariates, thereby reducing confounding. The study employed a large sample size, with real time data. While cross-sectional data cannot establish causality, our study provides an initial evaluation that identifies associations and trends. These findings can guide future longitudinal, interventional, and qualitative studies to more definitively examine causal relationships and further explore barriers and facilitators of virtual care among people living with HIV. |  |
| **Response to #2** | | |
| Please add a brief description of the burden of HIV in Canada with reference to population SES | We thank the reviewer for this comment. We have already provided references describing the Ontario HIV Treatment Cohort Study and the HIV burden in Ontario, which support the representativeness of our findings to the Ontario population. While some aspects of HIV care may differ in other provinces, generalizing these results to all of Canada would not serve the purpose of our study, which is to provide a detailed, context-specific evaluation of virtual care use and sociodemographic determinants in Ontario. These findings remain valuable for informing local public health strategies and can serve as a basis for future studies in other regions. However, there was a major discrepancy that we previously used CDC report 2023 for referencing of retention rates. Now we have made the change and used the Ontario HIV Epidemiology and Surveillance Initiative (OHESI) report, HIV Care Cascade in Ontario: Linkage to Care, In Care, On Antiretroviral Treatment, and Virally Suppressed, 2020. Toronto, ON: OHESI,  “In Ontario, 2020 data show that retention in HIV care varied across regions, with an overall 89.2% of people living with diagnosed HIV retained in care, and lower retention in some areas such as Northwestern Ontario (67.6%).” | **Page 6-7, lines 101-110** |
| Describe the category Men non-MSM | Thank you for this feedback. We acknowledge that the “Men non-MSM” category was not clearly defined. We have updated the terminology to “Heterosexual Men”, which is universally understood. Additionally, we have changed “Male:MSM” to “MSM” only, as this term is self-explanatory. These changes have been applied consistently in both Table 1 and Table 2, as well as throughout the manuscript. Moreover, we have used the term “hybrid” to replace the “combination of virtual and in-person care”, which is a term used consistently across the literature for people who access both in-person and virtual mediums. |  |
| **Response to Reviewer #3:** | | |
| 1.This manuscript examines the association between socioeconomic factors and the use of virtual care among people living with HIV (PLHIV) in a clinical cohort in Ontario, Canada. The authors assessed the use of virtual care in HIV management among infected patients, thereby providing valuable evidence for future studies and therapeutic interventions aimed at improving treatment adherence and outcomes in this population. The manuscript is generally well-organized, written in clear English, and engaging. The topic is highly relevant to public health practitioners and policymakers, as it provides a framework and rationale for future interventions using digital solutions to improve access to care. However, several sections require clarification and methodological improvements. | We sincerely thank the reviewer for their positive and encouraging comments. We appreciate their recognition of the study’s relevance and have addressed the suggested clarifications and methodological improvements in the revised manuscript. |  |
| 2.Methods section: It would be ideal for the authors to follow appropriate research guidelines in the presentation and reporting of their findings. A suitable guideline would be the Strengthening the Reporting of Observational Studies in Epidemiology (STROBE) statement. | We thank the reviewer for this helpful suggestion. We had followed the STROBE guidelines during the initial preparation of the manuscript; however, we have now carefully reviewed the checklist to ensure full compliance with all reporting requirements. We have added details on sample size determination and funding sources and have included the completed STROBE checklist as a supplementary file for reference. The changes are given below with specific page numbers.  “Sample size calculations:  As of December 31, 2022, 2155 individuals completed the OCS questionnaire across the ten OCS sites. The primary outcome is adherence to ART, with 692 participants with suboptimal adherence and 1293 participants with optimal adherence. We planned a study with 1930 subjects. The sample size resulted in 80% power to detect a difference of 20% or greater between participants with suboptimal and optimal [35]. The Type I error probability associated with the test of the null hypothesis is 0.05 for two-tailed chi-squared statistic. (PS: Power and Sample Size Calculation version 3.1.2, 2014 by W.D. Dupont & W.D. Plummer Jr).”  **Funding**  This study received no funding. | **Page 11, Lines 199-206**  **Page 23, Lines 338-339** |
| 3. Line 106: The information about the Ontario HIV Treatment Network Cohort Study in reference number 25 is not available in an open-access journal. I recommend briefly describing the cohort directly within the manuscript and citing the reference accordingly. | We thank the reviewer for this helpful feedback. While multiple studies have been published using data from the Ontario HIV Treatment Network Cohort Study (OCS), the most comprehensive description of the cohort is provided in Rourke et al. We have now added a brief summary of the OCS in the manuscript for clarity.  The revised text reads as follows:  “In brief, the source population of the Ontario HIV Treatment Network Cohort Study (OCS) includes people living with HIV aged 16 years and older who receive medical care at specialty clinics within Ontario’s publicly funded healthcare system. Participants are recruited from 15 sites, including hospital-based outpatient clinics and community-based practices. Clinical data from routine follow-up visits are obtained through manual or electronic medical record abstraction, linkage with Public Health Ontario Laboratories (PHOL), the sole provincial provider of HIV-related testing, and annual standardized interviews that capture sociodemographic, psychosocial, and behavioral information. Upon enrolment, participants complete a baseline questionnaire and subsequently an annual follow-up interview addressing demographics, employment, income, mental health, and substance use.” | **Page 6-7, Lines 102-114** |
| 4. Results Section:   P-values should be reported in the tables. I suggest using APA style for presenting the findings and including all relevant model parameters for the multinomial logistic regression analysis. Additionally, I recommend reporting p-values for all variables, especially those associated with the adjusted odds ratios. | Thank you for your feedback. We have presented the results using both unadjusted and adjusted estimates along with their corresponding confidence intervals and have bolded the significant findings to facilitate easy interpretation.  Due to space constraints, we were unable to provide full p-values or APA-style formatting in the tables. We believe this approach, used for multinomial regression, effectively conveys the key results while remaining concise. |  |
| 5. Discussion Section:   Several cited studies are not properly referenced.  Most of the reported findings have not been adequately discussed in the context of whether they support or contradict previous research. Their implications should also be addressed.  Please refer to the STROBE guidelines for further guidance. | We have addressed the reviewer’s comments by enhancing the discussion with relevant and up-to-date references and following the STROBE guidelines to discuss the other evidence, limitations, bias, and applicability of the findings. | **Page 20-22.** |
| 6. Acknowledgments Section:   The acknowledgments section is too long. It should be limited to a single paragraph. Please refer to published PLOS ONE articles for formatting examples. | We have summarized the acknowledgement. Though the original was provided by the OCS Team, we acknowledge that it's very long for a journal.  The revised version now reads as follows:  “The Ontario HIV Treatment Network Cohort Study (OCS) is led by Dr. Lawrence Mbuagbaw (Principal Investigator) with contributions from co-investigators and collaborators across multiple institutions, including Unity Health, the University of Toronto, McMaster University, CAMH, and several clinical and community health centres across Ontario. We gratefully acknowledge the people living with HIV who participate in the OCS, as well as the OCS Governance Committee, Scientific Steering Committee, and Indigenous Data Governance Circle for their guidance and oversight. We also thank all research staff, coordinators, and clinicians involved in data collection and management, as well as Public Health Ontario Laboratories for supporting data linkage. The OCS is funded by the Ontario Ministry of Health. The views expressed are those of the authors and do not necessarily reflect those of the Ontario HIV Treatment Network or Public Health Ontario. | **Page 23, Line 337-348** |
