# Supplementary material for: Socioeconomic determinants of virtual care use among people living with HIV in a clinical cohort in Ontario, Canada: A cross-sectional study
Source: PLoS One. 2026 Feb 19;21(2):e0326989. doi: 10.1371/journal.pone.0326989 (PMC12919790; doi:10.1371/journal.pone.0326989)
Supplement: Attachment — Submitted filename: Response_to_reviewers_auresp_2.docx [file pone.0326989.s007.docx]

**Nadia Rehman,**

PhD candidate,

Department of Health Research Methods, Evidence, and Impact,

1280 Main Street West,

Hamilton, ON L8S 4K1

Tel: 289-439-7433

Email: [rehmann@mcmaster.ca](mailto:rehmann@mcmaster.ca)

December 29th^th^, 2025

**Subject: Revised Submission – Manuscript ID PONE-D-25-31000**

Dear Dr. Jiawen and PLOS ONE Editorial Team,

Thank you for the opportunity to revise and resubmit our manuscript (PONE-D-25-31000), entitled *“Socioeconomic determinants of virtual care use among people living with HIV in a clinical cohort in Ontario, Canada: A cross-sectional study,”* for consideration in **PLOS ONE**.

We sincerely appreciate the support of the PLOS ONE editorial team and are grateful to the reviewers for their time, expertise, and constructive feedback. We have carefully addressed each comment and incorporated the suggested revisions to improve the clarity, methodological rigor, and overall quality of the manuscript.

Below, we provide a detailed, point-by-point response to all reviewer comments. A revised version of the manuscript, along with a marked-up version highlighting the changes, has been submitted for your review. We believe these revisions align with the journal’s standards and address the reviewers' concerns.

Thank you for your consideration. We look forward to your feedback.

Sincerely,

Nadia Rehman

| **Response to Reviewer # 1:** | | |
| --- | --- | --- |
| **Reviewer’s comments** | **Authors’ response** | **Page No.** |
| 4. Have the authors made all data underlying the findings in their manuscript fully available? (No) | We obtained the data for this study from the Ontario HIV Treatment Network Cohort Study (OCS). Because the cohort comprises individuals from a vulnerable population, people living with HIV who may also experience social and economic marginalization, access to the data is strictly controlled. The data are provided only following approval of a research protocol by the OCS governance and review committees, contingent upon formal data-sharing and confidentiality agreements. Under these agreements, the authors are not legally permitted to publicly distribute or share the raw dataset.  In accordance with PLOS ONE’s data availability and transparency requirements, we have revised the manuscript to ensure that all required information is clearly provided to enable other researchers to understand the data source and request access through appropriate channels. **1) A description of the data set and the third-party source.**  We have updated the Methods section to include a detailed description of the dataset and its third-party source. The text now reads s follows:  “We conducted a cross-sectional study using data collected in 2022 from participants enrolled in the OCS. The OCS is an ongoing, multisite clinical cohort of people living with HIV who are receiving HIV care in Ontario, Canada. Detailed descriptions of the cohort have been published previously [22]. Briefly, the source population of the OCS includes individuals aged 16 years and older living with HIV who receive medical care within Ontario’s publicly funded healthcare system. Participants are recruited from 15 HIV care sites across the province, including hospital-based outpatient clinics and community-based practices. The OCS integrates longitudinal clinical data with patient-reported information using standardized data collection procedures.  Clinical data are obtained through manual and electronic abstraction of medical records from routine follow-up visits and are supplemented by linkage with Public Health Ontario Laboratories, the sole provincial provider of HIV-related laboratory testing, including viral load measurements. These clinical data are complemented by standardized questionnaires administered at enrollment and annually thereafter, which collect detailed sociodemographic, socioeconomic, psychosocial, and behavioral information, including employment, income, mental health, substance use, and healthcare utilization [23].  For the present analysis, we used cross-sectional data from the 2022 OCS questionnaire, which included detailed measures of HIV care delivery modalities, including in-person and virtual care via telephone or video, as well as relevant demographic, socioeconomic, and clinical covariates.” **2) If applicable, verification of permission to use the data set:**  We have provided documentation verifying permission to use the dataset. Specifically, the confidentiality and data-sharing agreement between the authors and the Ontario HIV Treatment Network has been uploaded and is available under the file titled “OHTN_Confidentiality_Agreement.” 3**) Confirmation of whether the authors received any special privileges in accessing the data that other researchers would not have.**  The authors did not receive any special privileges in accessing the data. All data were obtained through the standard application and approval procedures established by the Ontario HIV Treatment Network (OHTN). Access to the Ontario HIV Treatment Network Cohort Study (OCS) data is governed by formal data-sharing agreements and ethical approvals, and the same process is available to any qualified researcher who submits a proposal for review and approval by OHTN. **4) All necessary contact information others would need to apply to gain access to the data.**  Access to the OCS data is available through a standardized application process. Interested researchers may request access by completing the Research Application Process (RAP) form available on the OHTN website and by contacting the OCS team directly at [ocs@ohtn.on.ca](mailto:ocs@ohtn.on.ca). This information is clearly stated in the Data Availability Statement, which reads:  “**Data Availability Statement**  The data used in this study were obtained from the Ontario HIV Treatment Network Cohort Study (OCS). Due to privacy and confidentiality agreements, the authors cannot share the dataset or raw data publicly. However, data access may be requested directly from the OHTN by submitting a [Research Application Process (RAP) form.](https://ohtncohortstudy.ca/research/) Requests can be made by emailing <ocs@ohtn.on.ca>.” | **Page 6-7, Lines 99-116**  **Page 24, Lines 378-382** |
| 1) We employed multinomial logistic regression to identify predictors of care mode: virtual, in-person, or a hybrid (virtual and in-person). How did the authors test the sensitivity to multicollinearity of the independent variables? How did the authors manage complex nonlinear interactions? | Thank you for this comment. Prior to fitting the multinomial logistic regression models, we conducted a series of diagnostic and preparatory analyses to assess model stability and specification.  Sensitivity to multicollinearity among independent variables was assessed using variance inflation factors (VIFs) derived from a linear proxy model that included the same set of covariates as the multinomial model. All VIF values were below 2, indicating very low collinearity and no evidence of multicollinearity sensitivity in parameter estimates. Corresponding tolerance values were greater than 0.5, further supporting the absence of problematic collinearity.  Regarding complex nonlinear interactions, interaction terms and higher-order nonlinear effects were not included in the final models. Model specification was guided by theoretical and clinical considerations. Based on prior literature and substantive knowledge, key variables, including age, depression severity, and viral load, were modelled as categorical variables to capture potentially nonlinear relationships. The only covariates retained as continuous variables were the Physical Component Summary (PCS-12) and Mental Component Summary (MCS-12) scores, which are validated continuous measures of health-related quality of life.  Plausibility and potential departures from linearity of continuous variables were assessed using graphical methods, including inspection of distributions, residual diagnostics from proxy regression models, and smoothed plots. These assessments did not indicate meaningful departures from linearity or improved model fit with interaction terms. Therefore, a parsimonious main-effects model was retained.  Influential observations were additionally assessed using Cook’s distance; although a small number exhibited elevated influence values, these reflected plausible participant characteristics rather than data errors, and their inclusion did not materially affect model estimates. Relevant clarifications have been added to the Statistical Analysis section. The Model Diagnostics and Specifications read as follows:  “Model Diagnostics and Specification  Prior to fitting the multinomial logistic regression models, diagnostic and preparatory analyses were conducted to assess model stability and specification. Multicollinearity among independent variables was evaluated using variance inflation factors (VIFs) derived from a linear proxy model that included the same set of covariates; all VIFs were below 2, indicating minimal multicollinearity among predictors. Influential observations were assessed using Cook’s distance; although a small number of observations exhibited elevated influence values, these reflected plausible participant characteristics and did not materially affect model estimates.  Theoretical and clinical considerations guided model specification. Variables such as age, depression severity, and viral load were a priori categorized to capture potentially nonlinear relationships supported by the existing literature. The only covariates modelled as continuous variables were the Physical Component Summary (PCS-12) and Mental Component Summary (MCS-12) scores, which are validated continuous measures of health-related quality of life. Plausibility and potential departures from linearity of continuous variables were evaluated using graphical methods, including inspection of distributions, residual diagnostics from proxy regression models, and smoothed plots. These assessments did not reveal meaningful departures from linearity or evidence that inclusion of interaction terms or higher-order nonlinear effects would improve model fit. Accordingly, a parsimonious main-effects multinomial logistic regression model was retained.” | **Page 11, Lines 195-211** |
| 2) We used a three-category multinomial logistic regression, with in-person care as the reference category, to identify independent correlates of virtual care use. How did the authors manage the limitation related to the hypothesis of independence of irrelevant alternatives? | Thank you for raising this important methodological consideration. We acknowledge the Independence of Irrelevant Alternatives (IIA) assumption inherent to multinomial logistic regression and took several steps to assess the robustness of our findings.  Although the Hausman–McFadden test did not indicate statistically significant violations of the Independence of Irrelevant Alternatives (IIA) assumption (p > 0.05), we recognize that this test has known limitations in applied settings, particularly in the presence of sparse cells and complex outcome structures. Therefore, rather than relying solely on this test, we conducted additional sensitivity analyses to evaluate the robustness of our findings.  Prior to model fitting, covariates with limited variability were identified. Housing situation and HIV related stigma showed sparse cell counts across care modality categories. When included in preliminary multinomial models, these variables resulted in numerical instability, including non convergence and undefined coefficient estimates. Consistent with known limitations of multinomial logistic regression under sparse data conditions, these covariates were excluded a priori from the final models to ensure stable estimation and interpretable inference.  Second, we conducted sensitivity analyses by excluding the hybrid care category and re-estimating the models using binary logistic regression comparing virtual versus in-person care. The direction and relative magnitude of associations were largely consistent with those observed in the multinomial models. Older age and privacy concerns were consistently associated with a lower likelihood of virtual care, whereas depression diagnosis, unemployment, higher income, and lower ART adherence were associated with a higher likelihood of virtual care across both modelling approaches. Although effect sizes differed in magnitude, as expected when comparing relative risk ratios and odds ratios, no meaningful discrepancies in interpretation were observed.  Finally, the three outcome categories: in-person care, virtual care, and hybrid care, were defined a priori and represent conceptually distinct modes of healthcare delivery, supporting the plausibility of the IIA assumption in this context. Taken together, these steps support the robustness of the primary findings despite the inherent limitations of the multinomial framework.  Relevant clarifications have been added to the Statistical Analysis section of the manuscript.  The revised methods section now reads as follows:  “The multinomial logistic regression model relies on the Independence of Irrelevant Alternatives (IIA) assumption. First, we applied the Hausman–McFadden specification test, which indicated no statistically significant violations of the IIA assumption (p > 0.05). However, given the known limitations of this test in finite samples and in models with correlated outcome categories, we additionally conducted a sensitivity analysis excluding the hybrid care category and fitted a binary logistic regression comparing virtual versus in-person care. The direction and magnitude of associations in the sensitivity analysis were consistent with those observed in the primary multinomial model, indicating that the substantive findings were not materially sensitive to potential violations of the IIA assumption. In addition, the outcome categories: in-person care, virtual care, and hybrid care, were defined a priori and represent conceptually distinct modes of healthcare delivery, supporting the appropriateness of the multinomial modelling framework. Covariates with sparse cell counts (housing and stigma) across outcome categories were excluded from the final models, as they led to numerical instability and undefined parameter estimates during multinomial model estimation.” | **Page 11-12, Lines 195-224** |
| 3) A paragraph on the implications of the results for practice and public health should be added. | We appreciate the reviewer for this feedback, we have added a paragraph addressing the implications of the findings:  **Implications for Practice and Public Health**  These findings support the continued use of hybrid HIV care models that integrate both virtual and in-person services. The absence of statistically significant differences in care modality use across age groups and in most access-related barriers suggests that virtual care did not systematically disadvantage older adults or individuals facing technological constraints within this publicly funded healthcare setting. The higher use of hybrid care among individuals with depression highlights the importance of flexible care delivery models that can accommodate fluctuating mental health needs while maintaining continuity of care. From a public health perspective, maintaining multiple modes of care delivery may help promote equitable access, support patient-centred care, and strengthen engagement in HIV services without exacerbating existing disparities. | **Page 22-23, lines 332-340** |
| **Editors’s Comments** | **Authors’ Response** | **Page No.** |
| **Assumption for multinomial regressions** | | |
| The most important aspect of the comment that I would like for you to focus on is the one regarding the independence assumption. Given that hybrid care is, conceptually, a mix of the other two categories, it cannot necessarily be considered independent of them, and patients would likely not divide evenly if this category were removed. This could be problematic for your multinomial logistic regression. There are tests that you can perform to assess the validity of this assumption for your analysis, which I invite you to consider completing as a part of your revision. | Addressed above under “Response to reviewers: Point 2.” |  |
| In regards to multicollinearity and nonlinear interactions. I am more interested in the multicollinearity part; given the inclusion of multiple SES variables, the study may benefit from assessment with VIFs. I think given your sample size and the complexity of the work required, an explicit exploration of non-linear interactions is not necessary. | Addressed above under response to reviewers: Point 1 |  |
| Lastly, I agree with the reviewer that a brief comment on public health implications is warranted. Discussions of UNAIDS target and prior studies are good, but suppose I am a clinic manager in Ontario, are there any specific take-aways for me after reading this article? | Addressed above under response to reviewer: Point 3 |  |
| **Journal requirements** | | |
| 1.Please ensure that your manuscript meets PLOS ONE's style requirements, including those for file naming. The PLOS ONE style templates can be found at https://journals.plos.org/plosone/s/file?id=wjVg/PLOSOne_formatting_sample_main_body.pdf and https://journals.plos.org/plosone/s/file?id=ba62/PLOSOne_formatting_sample_title_authors_affiliations.pdf | We have ensured that our manuscript meets PLOS ONE’s style formatting requirements. |  |
| 2. We noted in your submission details that a portion of your manuscript may have been presented or published elsewhere. “We would also like to highlight our recent publication in PLOS ONE, titled “Association between virtual visits and health outcomes of people living with HIV: A cross-sectional study,” based on the 2022 OCS cohort. While both manuscripts draw from the same cohort, they address distinct research questions. Given the differing focuses on aspects of virtual care, the findings are presented in separate publications.” Please clarify whether this [conference proceeding or publication] was peer-reviewed and formally published. If this work was previously peer-reviewed and published, in the cover letter please provide the reason that this work does not constitute dual publication and should be included in the current manuscript. | Thank you for the opportunity to clarify this point. We confirm that our related article, “Association between virtual visits and health outcomes of people living with HIV: A cross-sectional study,” was peer-reviewed and formally published in PLOS ONE in 2025 (Rehman et al., 2025).  Rehman, N., Mbuagbaw, L., Mertz, D., Muraca, G. M., Jones, A., & Ontario HIV Treatment Network Cohort Study. (2025). Association between virtual visits and health outcomes of people living with HIV: A cross-sectional study. *PLoS One*, *20*(5), e0315880.  Both manuscripts draw on data from the 2022 Ontario HIV Treatment Network Cohort Study (OCS); however, they address distinct research questions and outcomes. The previously published study examined the association between care modality (virtual, in-person, and hybrid) and clinical health outcomes, including antiretroviral therapy (ART) adherence, quality of life and viral load suppression. That analysis focused on whether different modes of care were associated with differences in clinical outcomes among people living with HIV.  In contrast, the current manuscript investigates the socioeconomic, structural, and demographic determinants of care modality use, with particular emphasis on barriers to access, patient characteristics, and contextual factors that influence whether individuals utilize virtual, in-person, or hybrid HIV care. Rather than evaluating health outcomes, this study seeks to understand who uses different modes of care and why, and whether disparities in access may underlie observed patterns of care utilization.  Given these clearly distinct objectives, analytic approaches, and outcome measures, the current manuscript does not constitute dual publication. Instead, it provides a complementary and policy-relevant contribution by contextualizing virtual care utilization within broader socioeconomic and access-related determinants, thereby extending the interpretation of virtual care use beyond clinical outcomes alone. |  |
| 3. For studies involving third-party data, we encourage authors to share any data specific to their analyses that they can legally distribute. PLOS recognizes, however, that authors may be using third-party data they do not have the rights to share. When third-party data cannot be publicly shared, authors must provide all information necessary for interested researchers to apply to gain access to the data. (https://journals.plos.org/plosone/s/data-availability#loc-acceptable-data-access-restrictions) For any third-party data that the authors cannot legally distribute, they should include the following information in their Data Availability Statement upon submission: 1) A description of the data set and the third-party source 2) If applicable, verification of permission to use the data set 3) Confirmation of whether the authors received any special privileges in accessing the data that other researchers would not have 4) All necessary contact information others would need to apply to gain access to the data | Addressed above under “Response to reviewers” point 4. |  |
| 4. One of the noted authors is a group or consortium “Ontario HIV Treatment Network Cohort Study”. In addition to naming the author group, please list the individual authors and affiliations within this group in the acknowledgments section of your manuscript. Please also indicate clearly a lead author for this group along with a contact email address.    If the reviewer comments include a recommendation to cite specific previously published works, please review and evaluate these publications to determine whether they are relevant and should be cited. There is no requirement to cite these works unless the editor has indicated otherwise. | Thank you for this request. We have updated the Acknowledgments section in accordance with OHTN guidance. The revised text is provided below.  “The OHTN Cohort Study Team consists of Dr. Lawrence Mbuagbaw (Principal Investigator; email: mbuagblc@mcmaster.ca), Department of Health Research Methods, Evidence, and Impact (HEI)  Chair and Principal Investigator; Ontario HIV Treatment Network Cohort Study (OCS); Dr. Anita Benoit (Co-Investigator), University of Toronto; Dr. Sergio Rueda, CAMH and University of Toronto; Dr. Gordon Arbess, Unity Health; Dr. Corinna Quan, Windsor Regional Hospital; Dr. Curtis Cooper, Ottawa General Hospital; Elizabeth Lavoie and Dr. Maheen Saeed, Byward Family Health Team; Dr. Mona Loutfy and Dr. David Knox, Maple Leaf Medical Clinic; Dr. Nisha Andany, Sunnybrook Health Sciences Centre; Dr. Sharon Walmsley, University Health Network; Dr. Michael Silverman, St. Joseph’s Health Care; Tammy Bourque, Health Sciences North; Dr. Marek Smieja, Hamilton Health Sciences Centre; Wangari Tharao, Women’s Health in Women’s Hands Community Health Centre; Holly Gauvin, Elevate NWO; Dr. Jorge Martinez-Cajas, Kingston Hotel Dieu Hospital; and Dr. Jeffrey Craig, Lakeridge Positive Care Clinic.  We gratefully acknowledge all of the people living with HIV who volunteer to participate in the OHTN Cohort Study. We also acknowledge the work and support of OCS Governance Committee (Aaron Bowerman, Adrian Betts, Barry Adam, Cornel Gray, Dane Record, Jasmine Cotnam, Jason Brophy, Mary Ndung’u, Rodney Rousseau, Ruth Cameron, YY Chen) OCS Scientific Steering Committee (Anita Benoit, Ann Burchell, Barry Adam, Curtis Cooper, David Brennan, Kelly O’Brien, Lance Mcready, Lawrence Mbuagbaw, Mona Loutfy, Pierre Giguere, Sean Hillier, Sergio Rueda (Chair), and Trevor Hart) and Indigenous Data Governance Circle (Meghan Young, Randy Jackson, Trevor Stratton). The OHTN also acknowledges the work of past Governance Committee and Scientific Steering Committee members.  We thank all interviewers, data collectors, research associates, coordinators, nurses, and physicians who provide data collection support. The authors also wish to thank OCS staff for data management, IT support, and study coordination: Lucia Light, Mustafa Karacam, Nahid Qureshi, and Tsegaye Bekele. The Ontario Ministry of Health supports the OHTN Cohort Study.  We also acknowledge the Public Health Laboratories, Public Health Ontario, for supporting record linkage with the HIV viral load database.  The OHTN Cohort Study is supported by the Ontario Ministry of Health and Long-Term Care.  The opinions, results and conclusions are those of the authors and no endorsement by the Ontario HIV Treatment Network or Public Health Ontario is intended or should be inferred.  Realize provided support and guidance for CAB recruitment, consultation on community engagement and study design. | **Page 25, Lines 386-415** |
| Please review your reference list to ensure that it is complete and correct. If you have cited papers that have been retracted, please include the rationale for doing so in the manuscript text, or remove these references and replace them with relevant current references. Any changes to the reference list should be mentioned in the rebuttal letter that accompanies your revised manuscript. If you need to cite a retracted article, indicate the article’s retracted status in the References list and also include a citation and full reference for the retraction notice. | We have checked our reference list and ensured that it is complete and correct. |  |
| To ensure your figures meet our technical requirements, please review our figure guidelines: https://journals.plos.org/plosone/s/figures | We have ensured that our figures meet the PLOS ONE’s figure technical requirements. |  |
| You may also use PLOS’s free figure tool, NAAS, to help you prepare publication quality figures: https://journals.plos.org/plosone/s/figures#loc-tools-for-figure-preparation. NAAS will assess whether your figures meet our technical requirements by comparing each figure against our figure specifications. | We used the PLOS’s figure tool, NAAS. We appreciate this support. We have uploaded the figures after final specification from NAAS. |  |

**Response to the reviewer’s comments:**

**4. Have the authors made all data underlying the findings in their manuscript fully available?**

The PLOS Data policy requires authors to make all data underlying the findings described in their manuscript fully available without restriction, with rare exceptions (please refer to the Data Availability Statement in the manuscript PDF file). The data should be provided as part of the manuscript or its supporting information, or deposited in a public repository. For example, in addition to summary statistics, the data points underlying mean, median, and variance measures should be available. If there are restrictions on publicly sharing data, e.g. participant privacy or use of data from a third party, those must be specified.

**Reviewer #1: No**

**Authors’ Response:**

We obtained the data for this study from the Ontario HIV Treatment Network Cohort Study (OCS). Because the cohort comprises individuals from a vulnerable population, people living with HIV who may also experience social and economic marginalization, access to the data is strictly controlled. The data are provided only following approval of a research protocol by the OCS governance and review committees, contingent upon formal data-sharing and confidentiality agreements. Under these agreements, the authors are not legally permitted to publicly distribute or share the raw dataset.

In accordance with PLOS ONE’s data availability and transparency requirements, we have revised the manuscript to ensure that all required information is clearly provided, enabling other researchers to understand the data source and request access through appropriate channels.

**1) A description of the data set and the third-party source.**

**Authors’ Response:**

We have updated the Methods section to include a detailed description of the dataset and its third-party source. Revised text for Page 6-7, Lines 99-116

“We conducted a cross-sectional study using data collected in 2022 from participants enrolled in the OCS. The OCS is an ongoing, multisite clinical cohort of people living with HIV who are receiving HIV care in Ontario, Canada. Detailed descriptions of the cohort have been published previously [22]. Briefly, the source population of the OCS includes individuals aged 16 years and older living with HIV who receive medical care within Ontario’s publicly funded healthcare system. Participants are recruited from 15 HIV care sites across the province, including hospital-based outpatient clinics and community-based practices. The OCS integrates longitudinal clinical data with patient-reported information using standardized data collection procedures.

Clinical data are obtained through manual and electronic abstraction of medical records from routine follow-up visits and are supplemented by linkage with Public Health Ontario Laboratories, the sole provincial provider of HIV-related laboratory testing, including viral load measurements. These clinical data are complemented by standardized questionnaires administered at enrollment and annually thereafter, which collect detailed sociodemographic, socioeconomic, psychosocial, and behavioral information, including employment, income, mental health, substance use, and healthcare utilization [23].

For the present analysis, we used cross-sectional data from the 2022 OCS questionnaire, which included detailed measures of HIV care delivery modalities, including in-person and virtual care via telephone or video, as well as relevant demographic, socioeconomic, and clinical covariates.”

**2) If applicable, verification of permission to use the data set:**

**Authors’ Response:**

We have provided documentation verifying permission to use the dataset. Specifically, the confidentiality and data-sharing agreement between the authors and the Ontario HIV Treatment Network has been uploaded and is available under the file titled “OHTN_Confidentiality_Agreement.”

**3) Confirmation of whether the authors received any special privileges in accessing the data that other researchers would not have.**

**Authors’ Response:**

The authors did not receive any special privileges in accessing the data. All data were obtained through the standard application and approval procedures established by the Ontario HIV Treatment Network (OHTN). Access to the Ontario HIV Treatment Network Cohort Study (OCS) data is governed by formal data-sharing agreements and ethical approvals, and the same process is available to any qualified researcher who submits a proposal for review and approval by OHTN.

**4) All necessary contact information others would need to apply to gain access to the data.**

**Authors’ Response:**

Access to the OCS data is available through a standardized application process. Interested researchers may request access by completing the Research Application Process (RAP) form available on the OHTN website and by contacting the OCS team directly at [ocs@ohtn.on.ca](mailto:ocs@ohtn.on.ca). This information is clearly stated in the Data Availability Statement, which reads:

Revised text for Page 24, Lines 378-382

“Data Availability Statement

The data used in this study were obtained from the Ontario HIV Treatment Network Cohort Study (OCS). Due to privacy and confidentiality agreements, the authors cannot share the dataset or raw data publicly. However, data access may be requested directly from the OHTN by submitting a Research Application Process (RAP) form. Requests can be made by emailing ocs@ohtn.on.ca.”

**6. Review Comments to the Author**

Reviewer #1: This manuscript provides a detailed overview of Socioeconomic determinants of virtual care use among people living with HIV in a clinical cohort in Ontario, Canada: A cross-sectional study. However, it contains a few errors. Consequently, the manuscript, in its current version, requires improvements in certain areas. Here are some detailed comments:

**1) We employed multinomial logistic regression to identify**

**predictors of care mode: virtual, in-person, or a hybrid (virtual and in-person).**

**How did the authors test the sensitivity to multicollinearity of the independent variables?**

**How did the authors manage complex nonlinear interactions?**

**Authors’ Response:**

Thank you for this comment. Prior to fitting the multinomial logistic regression models, we conducted a series of diagnostic and preparatory analyses to assess model stability and specification.

Sensitivity to multicollinearity among independent variables was assessed using variance inflation factors (VIFs) derived from a linear proxy model that included the same set of covariates as the multinomial model. All VIF values were below 2, indicating very low collinearity and no evidence of multicollinearity sensitivity in parameter estimates. Corresponding tolerance values were greater than 0.5, further supporting the absence of problematic collinearity.

Regarding complex nonlinear interactions, interaction terms and higher-order nonlinear effects were not included in the final models. Model specification was guided by theoretical and clinical considerations. Based on prior literature and substantive knowledge, key variables, including age, depression severity, and viral load, were modelled as categorical variables to capture potentially nonlinear relationships. The only covariates retained as continuous variables were the Physical Component Summary (PCS-12) and Mental Component Summary (MCS-12) scores, which are validated continuous measures of health-related quality of life.

Plausibility and potential departures from linearity of continuous variables were assessed using graphical methods, including inspection of distributions, residual diagnostics from proxy regression models, and smoothed plots. These assessments did not indicate meaningful departures from linearity or improved model fit with interaction terms. Therefore, a parsimonious main-effects model was retained.

Influential observations were additionally assessed using Cook’s distance; although a small number exhibited elevated influence values, these reflected plausible participant characteristics rather than data errors, and their inclusion did not materially affect model estimates. Relevant clarifications have been added to the Statistical Analysis section. The Model Diagnostics and Specifications read as follows:

“Model Diagnostics and Specification

Prior to fitting the multinomial logistic regression models, diagnostic and preparatory analyses were conducted to assess model stability and specification. Multicollinearity among independent variables was evaluated using variance inflation factors (VIFs) derived from a linear proxy model that included the same set of covariates; all VIFs were below 2, indicating minimal multicollinearity among predictors. Influential observations were assessed using Cook’s distance; although a small number of observations exhibited elevated influence values, these reflected plausible participant characteristics and did not materially affect model estimates.

Theoretical and clinical considerations guided model specification. Variables such as age, depression severity, and viral load were a priori categorized to capture potentially nonlinear relationships supported by the existing literature. The only covariates modelled as continuous variables were the Physical Component Summary (PCS-12) and Mental Component Summary (MCS-12) scores, which are validated continuous measures of health-related quality of life. Plausibility and potential departures from linearity of continuous variables were evaluated using graphical methods, including inspection of distributions, residual diagnostics from proxy regression models, and smoothed plots. These assessments did not reveal meaningful departures from linearity or evidence that inclusion of interaction terms or higher-order nonlinear effects would improve model fit. Accordingly, a parsimonious main-effects multinomial logistic regression model was retained.”

**2) We used a three-category multinomial logistic regression, with in-person care as the reference category, to identify independent correlates of virtual care use.**

**How did the authors manage the limitation related to the hypothesis of independence of irrelevant alternatives?**

**Authors’ Response:**

Thank you for raising this important methodological consideration. We acknowledge the Independence of Irrelevant Alternatives (IIA) assumption inherent to multinomial logistic regression and took several steps to assess the robustness of our findings.

Thank you for raising this important methodological consideration. We acknowledge the Independence of Irrelevant Alternatives (IIA) assumption inherent to multinomial logistic regression and took several steps to assess the robustness of our findings.

Although the Hausman–McFadden test did not indicate statistically significant violations of the Independence of Irrelevant Alternatives (IIA) assumption (p > 0.05), we recognize that this test has known limitations in applied settings, particularly in the presence of sparse cells and complex outcome structures. Therefore, rather than relying solely on this test, we conducted additional sensitivity analyses to evaluate the robustness of our findings.

Prior to model fitting, covariates with limited variability were identified. Housing situation and HIV related stigma showed sparse cell counts across care modality categories. When included in preliminary multinomial models, these variables resulted in numerical instability, including non convergence and undefined coefficient estimates. Consistent with known limitations of multinomial logistic regression under sparse data conditions, these covariates were excluded a priori from the final models to ensure stable estimation and interpretable inference.

Second, we conducted sensitivity analyses by excluding the hybrid care category and re-estimating the models using binary logistic regression comparing virtual versus in-person care. The direction and relative magnitude of associations were largely consistent with those observed in the multinomial models. Older age and privacy concerns were consistently associated with a lower likelihood of virtual care, whereas depression diagnosis, unemployment, higher income, and lower ART adherence were associated with a higher likelihood of virtual care across both modelling approaches. Although effect sizes differed in magnitude, as expected when comparing relative risk ratios and odds ratios, no meaningful discrepancies in interpretation were observed.

Finally, the three outcome categories: in-person care, virtual care, and hybrid care, were defined a priori and represent conceptually distinct modes of healthcare delivery, supporting the plausibility of the IIA assumption in this context. Taken together, these steps support the robustness of the primary findings despite the inherent limitations of the multinomial framework.

Relevant clarifications have been added to the Statistical Analysis section of the manuscript.

Multinomial logistic regression assumes independence of irrelevant alternatives (IIA), in which the relative odds of selecting one outcome category over another are independent of the presence of other alternatives. The revised methods section now reads as follows:

Revised methods section, pages 11-12, Lines 195-224.

“The multinomial logistic regression model relies on the Independence of Irrelevant Alternatives (IIA) assumption. First, we applied the Hausman–McFadden specification test, which indicated no statistically significant violations of the IIA assumption (p > 0.05). However, given the known limitations of this test in finite samples and in models with correlated outcome categories, we additionally conducted a sensitivity analysis excluding the hybrid care category and fitted a binary logistic regression comparing virtual versus in-person care. The direction and magnitude of associations in the sensitivity analysis were consistent with those observed in the primary multinomial model, indicating that the substantive findings were not materially sensitive to potential violations of the IIA assumption. In addition, the outcome categories: in-person care, virtual care, and hybrid care, were defined a priori and represent conceptually distinct modes of healthcare delivery, supporting the appropriateness of the multinomial modelling framework. Covariates with sparse cell counts (housing and stigma) across outcome categories were excluded from the final models, as they led to numerical instability and undefined parameter estimates during multinomial model estimation.”

**3) A paragraph on the implications of the results for practice and public health should be added.**

**Authors’ Response:**

**Revised discussion on implications of the findings: Page 22-23, lines 332-340**

**Implications for Practice and Public Health**

These findings support the continued use of hybrid HIV care models that integrate both virtual and in-person services. The absence of statistically significant differences in care modality use across age groups and in most access-related barriers suggests that virtual care did not systematically disadvantage older adults or individuals facing technological constraints within this publicly funded healthcare setting. The higher use of hybrid care among individuals with depression highlights the importance of flexible care delivery models that can accommodate fluctuating mental health needs while maintaining continuity of care. From a public health perspective, maintaining multiple modes of care delivery may help promote equitable access, support patient-centred care, and strengthen engagement in HIV services without exacerbating existing disparities.

**Journal requirements:
When submitting your revision, we need you to address these additional requirements.
1.Please ensure that your manuscript meets PLOS ONE's style requirements, including those for file naming. The PLOS ONE style templates can be found at**
https://journals.plos.org/plosone/s/file?id=wjVg/PLOSOne_formatting_sample_main_body.pdf and
<https://journals.plos.org/plosone/s/file?id=ba62/PLOSOne_formatting_sample_title_authors_affiliations.pdf>

**Authors’ Response:**

We have ensured that our manuscript meets PLOS ONE’s style formatting requirements. **2. We noted in your submission details that a portion of your manuscript may have been presented or published elsewhere. “We would also like to highlight our recent publication in PLOS ONE, titled “Association between virtual visits and health outcomes of people living with HIV: A cross-sectional study,” based on the 2022 OCS cohort. While both manuscripts draw from the same cohort, they address distinct research questions. Given the differing focuses on aspects of virtual care, the findings are presented in separate publications.” Please clarify whether this [conference proceeding or publication] was peer-reviewed and formally published. If this work was previously peer-reviewed and published, in the cover letter please provide the reason that this work does not constitute dual publication and should be included in the current manuscript.**

**Authors’ Response:**

Thank you for the opportunity to clarify this point. We confirm that our related article, “Association between virtual visits and health outcomes of people living with HIV: A cross-sectional study,” was peer-reviewed and formally published in PLOS ONE in 2025 (Rehman et al., 2025).

Rehman, N., Mbuagbaw, L., Mertz, D., Muraca, G. M., Jones, A., & Ontario HIV Treatment Network Cohort Study. (2025). Association between virtual visits and health outcomes of people living with HIV: A cross-sectional study. *PLoS One*, *20*(5), e0315880.

Both manuscripts draw on data from the 2022 Ontario HIV Treatment Network Cohort Study (OCS); however, they address distinct research questions and outcomes. The previously published study examined the association between care modality (virtual, in-person, and hybrid) and clinical health outcomes, including antiretroviral therapy (ART) adherence and viral load suppression. That analysis focused on whether different modes of care were associated with differences in clinical outcomes among people living with HIV.

In contrast, the current manuscript investigates the socioeconomic, structural, and demographic determinants of care modality use, with a particular emphasis on barriers to access, patient characteristics, and contextual factors influencing whether individuals utilize virtual, in-person, or hybrid HIV care. Rather than evaluating health outcomes, this study seeks to understand who uses different modes of care and why, and whether disparities in access may underlie observed patterns of care utilization.

Given these clearly distinct objectives, analytic approaches, and outcome measures, the current manuscript does not constitute dual publication. Instead, it provides a complementary and policy-relevant contribution by contextualizing virtual care utilization within broader socioeconomic and access-related determinants, thereby extending the interpretation of virtual care use beyond clinical outcomes alone. **3. For studies involving third-party data, we encourage authors to share any data specific to their analyses that they can legally distribute. PLOS recognizes, however, that authors may be using third-party data they do not have the rights to share. When third-party data cannot be publicly shared, authors must provide all information necessary for interested researchers to apply to gain access to the data. (https://journals.plos.org/plosone/s/data-availability#loc-acceptable-data-access-restrictions)**
For any third-party data that the authors cannot legally distribute, they should include the following information in their Data Availability Statement upon submission:
1) A description of the data set and the third-party source
2) If applicable, verification of permission to use the data set
3) Confirmation of whether the authors received any special privileges in accessing the data that other researchers would not have
4) All necessary contact information others would need to apply to gain access to the data

**Authors’ Response:**

Provided as a part of the response to the reviewer.  **4. One of the noted authors is a group or consortium, “Ontario HIV Treatment Network Cohort Study”. In addition to naming the author group, please list the individual authors and affiliations within this group in the acknowledgments section of your manuscript. Please also indicate clearly a lead author for this group along with a contact email address.**

**Authors’ Response:**

Thank you for this request. We have updated the Acknowledgments section in accordance with OHTN guidance. The revised text is provided below (Page 25, Lines 386-415).

“The OHTN Cohort Study Team consists of Dr. Lawrence Mbuagbaw (Principal Investigator; email: mbuagblc@mcmaster.ca), Department of Health Research Methods, Evidence, and Impact (HEI)

Chair and Principal Investigator; Ontario HIV Treatment Network Cohort Study (OCS); Dr. Anita Benoit (Co-Investigator), University of Toronto; Dr. Sergio Rueda, CAMH and University of Toronto; Dr. Gordon Arbess, Unity Health; Dr. Corinna Quan, Windsor Regional Hospital; Dr. Curtis Cooper, Ottawa General Hospital; Elizabeth Lavoie and Dr. Maheen Saeed, Byward Family Health Team; Dr. Mona Loutfy and Dr. David Knox, Maple Leaf Medical Clinic; Dr. Nisha Andany, Sunnybrook Health Sciences Centre; Dr. Sharon Walmsley, University Health Network; Dr. Michael Silverman, St. Joseph’s Health Care; Tammy Bourque, Health Sciences North; Dr. Marek Smieja, Hamilton Health Sciences Centre; Wangari Tharao, Women’s Health in Women’s Hands Community Health Centre; Holly Gauvin, Elevate NWO; Dr. Jorge Martinez-Cajas, Kingston Hotel Dieu Hospital; and Dr. Jeffrey Craig, Lakeridge Positive Care Clinic.

We gratefully acknowledge all of the people living with HIV who volunteer to participate in the OHTN Cohort Study. We also acknowledge the work and support of OCS Governance Committee (Aaron Bowerman, Adrian Betts, Barry Adam, Cornel Gray, Dane Record, Jasmine Cotnam, Jason Brophy, Mary Ndung’u, Rodney Rousseau, Ruth Cameron, YY Chen) OCS Scientific Steering Committee (Anita Benoit, Ann Burchell, Barry Adam, Curtis Cooper, David Brennan, Kelly O’Brien, Lance Mcready, Lawrence Mbuagbaw, Mona Loutfy, Pierre Giguere, Sean Hillier, Sergio Rueda (Chair), and Trevor Hart) and Indigenous Data Governance Circle (Meghan Young, Randy Jackson, Trevor Stratton). The OHTN also acknowledges the work of past Governance Committee and Scientific Steering Committee members.

We thank all interviewers, data collectors, research associates, coordinators, nurses, and physicians who provide data collection support. The authors also wish to thank OCS staff for data management, IT support, and study coordination: Lucia Light, Mustafa Karacam, Nahid Qureshi, and Tsegaye Bekele. The Ontario Ministry of Health supports the OHTN Cohort Study.

We also acknowledge the Public Health Laboratories, Public Health Ontario, for supporting record linkage with the HIV viral load database.

The OHTN Cohort Study is supported by the Ontario Ministry of Health and Long-Term Care.

The opinions, results and conclusions are those of the authors and no endorsement by the Ontario HIV Treatment Network or Public Health Ontario is intended or should be inferred.

Realize provided support and guidance for CAB recruitment, consultation on community engagement and study design.  **Please review your reference list to ensure that it is complete and correct. If you have cited papers that have been retracted, please include the rationale for doing so in the manuscript text, or remove these references and replace them with relevant current references. Any changes to the reference list should be mentioned in the rebuttal letter that accompanies your revised manuscript. If you need to cite a retracted article, indicate the article’s retracted status in the References list and also include a citation and full reference for the retraction notice.**

**Authors’ Response:**

We have checked our reference list and ensured that it is complete and correct.

**To ensure your figures meet our technical requirements, please review our figure guidelines:** [**https://journals.plos.org/plosone/s/figures**](https://journals.plos.org/plosone/s/figures)**.**

We have ensured that our figures meet the PLOS ONE’s figure technical requirements.

**You may also use PLOS’s free figure tool, NAAS, to help you prepare publication quality figures: https://journals.plos.org/plosone/s/figures#loc-tools-for-figure-preparation.**

**Authors’ Response:**

We used the PLOS’s figure tool, NAAS. We appreciate this support. We have uploaded the figures after final specification from NAAS.
